# Clinical Significance of Pathological Lymph Node Status in Prostate Cancer Patients Undergoing Radical Prostatectomy: A 25‐Year Institutional Cohort Study

**DOI:** 10.1155/aiu/1399937

**Published:** 2026-08-18

**Authors:** Tomoyuki Shimabukuro, Chietaka Ohmi, Koji Shiraishi

**Affiliations:** ^1^ Department of Urology, Ube Central Hospital, Ube, Yamaguchi, Japan; ^2^ Department of Urology, Graduate School of Medicine, Yamaguchi University, Ube, Yamaguchi, Japan, yamaguchi-u.ac.jp

**Keywords:** biochemical recurrence, overall survival, pelvic lymph node metastases, prognostic significance, radical prostatectomy

## Abstract

**Background:**

While the therapeutic value of pelvic lymph node dissection (PLND) remains controversial, the pathological lymph node status itself may provide important prognostic information after radical prostatectomy (RP).

**Methods:**

Among 610 consecutive patients who underwent RP between January 2000 and December 2019, with follow‐up through December 2024, we retrospectively analyzed 445 patients with intermediate‐ or high‐risk disease according to the D’Amico classification who underwent PLND. Patients were stratified according to the pathological lymph node status into pN0 (*n* = 416) and pN1 (*n* = 29) groups. The primary endpoint was overall survival (OS), and secondary endpoints included biochemical recurrence (BCR) and the prognostic significance of pathological lymph node status.

**Results:**

The median age of the cohort was 68 years. BCR occurred in 26% of the pN0 patients, with a median time to BCR of 29 months, and in 41% of pN1 patients, with a median time to BCR of 47 months. During follow‐up, 38 all‐cause deaths (9.1%) occurred in the pN0 group and 9 (31.0%) in the pN1 group. Kaplan–Meier analysis showed no significant difference in BCR‐free survival between groups, whereas OS was significantly worse in the pN1 group (hazard ratio: 2.768; 95% confidence interval: 1.315–5.829; *p* = 0.007). Ten‐year OS rates were 94% for pN0 patients and 74% for pN1 patients. Multivariable analysis identified the pN1 status and postoperative Gleason score of 7 as independent prognostic factors for OS.

**Conclusions:**

Pathological lymph node positivity is a strong independent predictor of inferior OS in patients undergoing RP for localized prostate cancer. These findings underscore the need for tailored postoperative management strategies in patients with node‐positive disease.

## 1. Introduction

Radical prostatectomy (RP) remains one of the most widely employed definitive treatment modalities for patients with localized or locally advanced prostate cancer (PCa). Among men undergoing RP, the presence of lymph node metastasis has consistently been associated with adverse oncological outcomes, including an increased risk of biochemical recurrence (BCR) and cancer‐specific mortality. Accurate identification of patients harboring nodal metastases, therefore, has important implications for risk stratification, postoperative management, and the selection of adjuvant therapies.

While the therapeutic value of extended pelvic lymph node dissection (ePLND) remains controversial, pathological lymph node status itself may provide important prognostic information after RP. In the contemporary prostate‐specific antigen (PSA) era, no randomized or high‐quality observational studies have conclusively demonstrated that ePLND improves prostate cancer‐specific or overall survival (OS) [1–4]. Reflecting this uncertainty, the National Comprehensive Cancer Network (NCCN) guidelines do not incorporate the lymph node status into their risk classification system for localized disease [5]. Possible explanations for these inconclusive findings include the limited sample size, insufficient follow‐up duration, variability in surgical templates, or uncertainty regarding the true primary landing zones of prostatic lymphatic drainage [6]. Given the increased perioperative morbidity associated with more extensive lymphadenectomy, the potential risks of ePLND may outweigh its benefits for many men with low‐risk disease in current clinical practice [7].

Beyond surgical considerations, an equally important but less explored issue concerns the biological function of lymph nodes themselves. Lymph nodes play a central role in antitumor immunity, serving as critical sites for antigen presentation and immune cell activation. Nevertheless, metastatic cancer cells frequently evade immune surveillance and establish secondary growth within lymphatic tissue. Recently, a provocative study by Terasaki and colleagues provided novel mechanistic insights into this process [8]. Their work demonstrated that cancer cells can hijack mitochondria from surrounding immune cells within the lymph node microenvironment. This mitochondrial depletion impairs antigen presentation, costimulatory signaling, and the cytotoxic activity of natural killer and CD8^+^ T cells, thereby facilitating immune escape and metastatic progression. These findings suggest that the mere extent or anatomical coverage of lymph node dissection may be less important than the underlying biological interactions between cancer cells and immune components within lymphatic tissue.

Against this background, a clearer understanding of the true clinical significance of lymph node involvement after RP is urgently needed. By analyzing our long‐term institutional experience, we sought to compare oncological outcomes—including BCR and OS—between patients with pathologically node‐positive and node‐negative disease. Our aim was to elucidate the incidence, clinicopathological characteristics, and prognostic impact of lymph node metastasis following RP.

Using our 25‐year institutional cohort, this study aims to refine postoperative risk stratification and clarify the prognostic significance of pathological lymph node involvement following RP. Improved understanding of nodal disease may help optimize postoperative management strategies, including the selective use of adjuvant and salvage therapies, while balancing oncological efficacy against overtreatment, quality‐of‐life deterioration, and financial burden in routine clinical practice.

## 2. Methods

### 2.1. Study Patients

Between January 2000 and December 2019, a total of 1278 patients with pathologically confirmed PCa were treated at Yamaguchi University Hospital and Ube Central Hospital. Among these, 610 consecutive patients underwent RP. Open retropubic RP (ORRP, *n* = 267) was primarily performed during the early study period, whereas robot‐assisted RP (RARP, *n* = 343) was introduced after September 2012. From this surgical cohort, we retrospectively analyzed 445 patients (ORRP, *n* = 202, and RARP, *n* = 243) classified as having intermediate‐ or high‐risk disease according to the D’Amico risk classification who underwent RP with PLND and had complete follow‐up data available through December 2024. Patients with low‐risk disease who did not undergo PLND and those with insufficient follow‐up data were excluded from the present analysis. Patients were stratified into two groups based on the pathological lymph node status: lymph node negative (pN0, *n* = 416) and lymph node positive (pN1, *n* = 29) (Table 1). The primary endpoint of this study was to identify independent prognostic factors for BCR‐free survival and OS according to the pN status. Secondary endpoints included the evaluation of clinicopathological characteristics associated with lymph node positivity.

**TABLE 1:** Baseline demographic and clinicopathological characteristics of patients with and without pathological lymph node metastasis.

	All	pN1	pN0	*p* value
	(*n* = 445)	(*n* = 29)	(*n* = 416)	
Age at pathological diagnosis, y				0.401
< 68	212	16	196	
≥ 68	233	13	220	
Baseline PSA level (ng/mL)				0.120
≤ 10.0	226	13	213	
10.1–20.0	154	8	146	
> 20.0	65	8	57	
D’Amico risk classification				0.017
Intermediate risk	233	9	224	
High risk	212	20	192	
Pathological T stage				< 0.001
≤ T2	309	10	299	
≥ T3	126	19	107	
Postoperative Gleason score				< 0.001
≤ 6	62	1	61	
7	258	11	247	
≥ 8	94	14	80	
Resected lymph nodes number				0.531
Median	9	11	9	
IQR	6–13	8–14	6–13	
Follow‐up characteristics				
Follow‐up months				0.865
Median	105.5	97.0	106.0	
IQR	78.0–141.0	70.3–155.8	78.0–140.0	
Biochemical recurrence: no. (%)	121 (27.2)	12 (41.4)	109 (26.2)	0.055
Time to BCR (months)				0.386
Median	29.0	47.0	28.5	
IQR	12.0–70.0	10.0–90.5	12.0–64.5	
Mortality: no. (%)				
All‐cause mortality	47 (10.6)	9 (31.0)	38 (9.1)	< 0.001
PCa‐specific mortality	6 (1.3)	4 (13.8)	2 (0.5)	< 0.001

*Note:* BCR, biochemical recurrence; PCa, prostate cancer; IQR, interquartile range; y, years.

Abbreviation: PSA, prostate‐specific antigen.

### 2.2. Diagnostic Procedures

Diagnostic evaluation and clinical staging were performed as previously described [9]. Tumor staging was based on the Tumor‐Node‐Metastasis (TNM) classification system, and risk groups were determined according to the D’Amico criteria. BCR after RP was defined as a serum PSA concentration ≥ 0.2 ng/mL, confirmed by a subsequent measurement [10].

### 2.3. Eligibility Criteria for RP and PLND

Patients were considered eligible for RP if they met the following criteria [9]:

• Age ≤ 75 years with an estimated life expectancy of at least 10 years
• Eastern Cooperative Oncology Group performance status ≤ 1
• Initial serum PSA level < 20 ng/mL
• Clinical stage ≤ T2 without evidence of regional lymph node or distant metastases (N0M0)

Patients with uncontrolled glaucoma, severe cardiovascular disease, intracranial lesions, or severe obstructive pulmonary disease were excluded from surgical treatment. In low‐to‐intermediate‐risk cases, multiparametric magnetic resonance (MRI) imaging findings were considered, and nerve‐sparing techniques were actively employed when oncologically appropriate. PLND was omitted in low‐risk cases.

In high‐risk cases, particularly those with a Gleason score ≥ 9, RP combined with extended PLND was performed. The extended PLND template included the obturator, internal and external iliac, and common iliac lymph node regions.

For patients with pathologically confirmed lymph node metastases, adjuvant androgen deprivation therapy (ADT) was initiated. The indication and timing of postoperative salvage radiotherapy were determined at the discretion of the treating physicians according to postoperative PSA kinetics and patient condition.

### 2.4. Data Collection

Clinical, pathological, and follow‐up data were retrospectively extracted from written and electronic medical records and, when necessary, supplemented by direct patient interviews. All data were reviewed using a standardized data collection form by two independent investigators to ensure accuracy and consistency. Any discrepancies were resolved by consensus [11]. The median follow‐up duration was 105.5 months.

### 2.5. Study Approval

This study was approved by the Institutional Review Board of the Yamaguchi University School of Medicine (approval number: H2020‐229) and by the Ethics Committee of Ube Central Hospital (approval number: 70160502).

### 2.6. Statistical Analysis

All statistical analyses were performed using StatView Version 4.5 (SAS, Cary, NC, USA). Associations between clinicopathological variables and lymph node status were evaluated using Fisher’s exact probability test for categorical variables and the Mann–Whitney *U* test for continuous variables. OS was estimated using the Kaplan–Meier method, and survival curves were compared using the log‐rank test. Univariable and multivariable Cox proportional hazards regression analyses were conducted to identify prognostic factors associated with OS. Variables found to be significant in univariable analyses were subsequently entered into the multivariable model.

All statistical tests were two‐sided, and a *p* value < 0.05 was considered statistically significant.

## 3. Results

### 3.1. Lymph Node Metastasis

**FIGURE 1:**
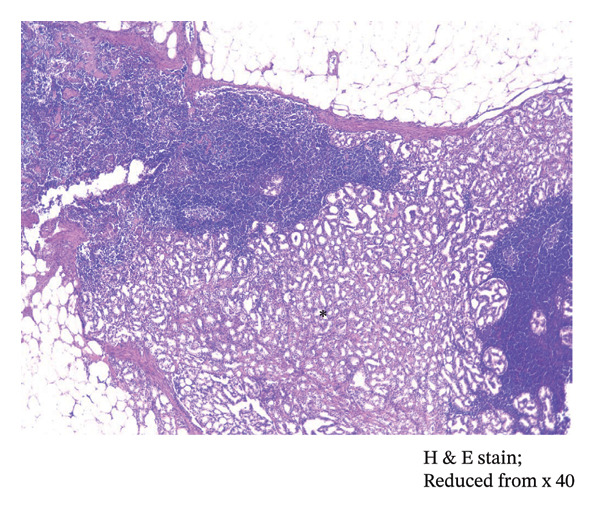
Histopathological findings of lymph node metastasis from prostate adenocarcinoma with a Gleason score of 9 in the left obturator lymph node (pT3a pN1, ly1, v0, pn1, sv0). Metastatic cancer cells are indicated by an asterisk (∗).

Figure 1 illustrates lymph node metastasis of adenocarcinoma with a Gleason score of 9 identified in the left obturator lymph node. Metastatic tumor cells extensively infiltrated the lymph node.

### 3.2. Baseline and Follow‐Up Characteristics

Baseline and follow‐up characteristics of the 445 patients, stratified by pathological lymph node status, are summarized in Table 1. The median age (interquartile range [IQR]) was 68 years (64–71) in the pN0 group and 67 years (61–71) in the pN1 group. Age distribution, baseline PSA levels, and the number of resected lymph nodes were comparable between the two groups.

Significant differences were observed in D’Amico risk classification, pathological T stage, and postoperative Gleason score. Patients in the pN1 group were more likely to belong to higher D’Amico risk categories, have advanced pathological T stages, and exhibit higher Gleason scores than those in the pN0 group.

The median follow‐up duration was 106 months (78–140) in the pN0 group and 97 months (70–156) in the pN1 group. In the pN1 group, all 29 patients received adjuvant ADT and 5 of these patients also received salvage radiotherapy. BCR occurred in 26% of patients in the pN0 group, with a median time to recurrence of 29 months, and in 41% of patients in the pN1 group, with a median time to recurrence of 47 months. During follow‐up, 38 all‐cause deaths (9.1%) and 2 PCa‐specific deaths (0.5%) were observed in the pN0 group. In contrast, the pN1 group experienced 9 all‐cause deaths (31.0%) and 4 PCa‐specific deaths (13.8%).

### 3.3. Treatment Sequences

**FIGURE 2:**
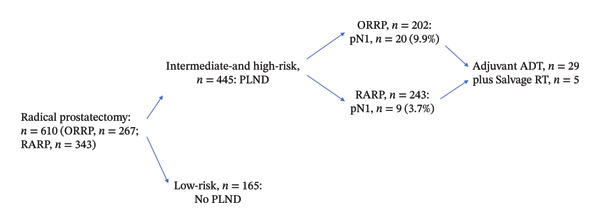
Flowchart of patient selection and treatment sequences in the study cohort. Abbreviations: ORRP, open retropubic radical prostatectomy; RARP, robot‐assisted radical prostatectomy; PLND, pelvic lymph node dissection: ADT, androgen deprivation therapy; RT, radiotherapy.

Figure 2 illustrates the treatment sequences and postoperative management strategies in the study cohort. Among 610 consecutive patients who underwent RP between 2000 and 2019, 445 intermediate‐ or high‐risk patients who underwent PLND and had complete follow‐up data were included in the final analysis. Surgical approaches included ORRP and RARP.

### 3.4. BCR‐Free Survival in Two Groups

**FIGURE 3:**
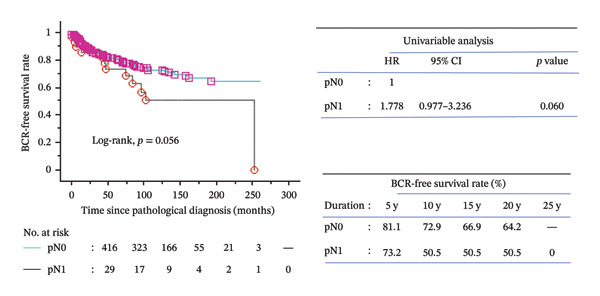
Kaplan–Meier curves of biochemical recurrence–free survival in patients treated with radical prostatectomy stratified by the pathological lymph node status. Abbreviations: BCR, biochemical recurrence; HR, hazard ratio; 95% CI, 95% confidence interval; y, years.

Kaplan–Meier curves for BCR‐free survival are shown in Figure 3. Although BCR‐free survival tended to be lower in the pN1 group than that in the pN0 group, the difference did not reach statistical significance. This finding may partly reflect the routine use of adjuvant ADT and salvage radiotherapy in patients with pN1 disease.

The 10‐year BCR‐free survival rates were 73% in the pN0 group and 51% in the pN1 group.

### 3.5. Prognostic Factors for BCR‐Free Survival

Cox proportional hazards regression analysis was performed to identify prognostic factors associated with BCR‐free survival (Table 2). In univariable analysis, a baseline PSA level > 20.0 ng/mL and pathological T stage ≥ T3 were significantly associated with poorer BCR‐free survival. In the multivariable analysis, a baseline PSA level > 20.0 ng/mL remained the only independent prognostic factor for BCR‐free survival.

**TABLE 2:** Univariable and multivariable cox proportional hazards analyses of risk factors associated with biochemical recurrence–free survival in the study cohort.

	Univariable			Multivariable		
	HR	95% CI	*p* value	HR	95% CI	*p* value
Age, y						
< 68	1			1		
≥ 68	1.049	0.731–1.503	0.797	0.984	0.674–1.435	0.932
Baseline PSA level (ng/mL)						
≤ 10	1			1		
10.1–20.0	1.107	0.725–1.690	0.637	1.164	0.744–1.822	0.506
> 20.0	2.811	1.801–4.387	< 0.001	3.971	2.152–7.327	< 0.001
D’Amico risk classification						
Intermediate risk	1			1		
High risk	1.336	0.931–1.917	0.116	0.662	0.394–1.114	0.120
Pathological T stage						
≤ 2	1			1		
≥ 3	1.486	1.020–2.166	0.039	1.082	0.710–1.648	0.715
Pathological N stage						
0	1			1		
1	1.778	0.977–3.236	0.060	1.217	0.628–2.360	0.561
Postoperative Gleason score						
≤ 6	1			1		
7	0.805	0.486–1.334	0.399	0.949	0.566–1.591	0.842
≥ 8	1.348	0.767–2.370	0.299	1.418	0.758–2.652	0.274

*Note:* 95% CI, 95% confidence interval; y, years.

Abbreviations: HR, hazard ratio; PSA, prostate‐specific antigen.

### 3.6. OS

**FIGURE 4:**
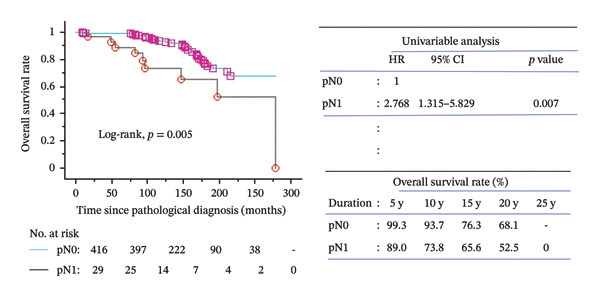
Kaplan–Meier curves of overall survival in patients treated with radical prostatectomy stratified by the pathological lymph node status. Abbreviations: HR, hazard ratio; 95% CI, 95% confidence interval; y, years.

Kaplan–Meier curves for OS are presented in Figure 4. Despite postoperative multimodal treatment, OS remained significantly worse in the pN1 group than that in the pN0 group. Pathological lymph node positivity was associated with a hazard ratio (HR) of 2.768 (95% confidence interval [CI], 1.315–5.829; *p* = 0.007). The 10‐year OS rates were 94% for the pN0 group and 74% for the pN1 group.

### 3.7. Prognostic Factors for OS

Cox regression analysis was conducted to identify prognostic factors for OS (Table 3). In univariable analysis, advanced age, high‐risk D’Amico classification, and pathological lymph node positivity (pN1) were significantly associated with poorer OS. In multivariable analysis, pN1 and postoperative Gleason score of 7 were identified as independent prognostic factors for OS. The lower HR of higher postoperative Gleason scores may partly reflect the use of perioperative ADT and adjuvant radiotherapy in patients with higher Gleason score disease.

**TABLE 3:** Univariable and multivariable cox proportional hazards analyses of risk factors associated with overall survival in the study cohort.

	Univariable			Multivariable		
	HR	95% CI	*p* value	HR	95% CI	*p* value
Age, y						
< 68	1			1		
≥ 68	2.244	1.241–4.059	0.008	3.993	0.930–17.132	0.062
Baseline PSA level (ng/mL)						
≤ 10	1			1		
10.1–20.0	1.133	0.567–2.267	0.723	2.804	0.486–16.198	0.249
> 20.0	1.961	0.942–4.082	0.072	2.764	0.475–16.090	0.258
D’Amico risk classification						
Intermediate risk	1			1		
High risk	1.841	1.034–3.278	0.038	2.839	0.408–19.745	0.292
Pathological T stage						
≤ 2	1			1		
≥ 3	1.759	0.978–3.167	0.060	0.836	0.225–3.107	0.789
Pathological N stage						
0	1			1		
1	2.768	1.315–5.829	0.007	19.024	2.497–144.925	0.005
Postoperative Gleason score						
≤ 6	1			1		
7	0.619	0.307–1.247	0.179	0.112	0.018–0.690	0.018
≥ 8	1.340	0.590–3.044	0.485	0.322	0.045–2.301	0.258
Time to BCR (median = 29 months)						
Longer	1			1		
Shorter	1.211	0.464–3.164	0.696	0.943	0.239–3.714	0.933

*Note:* BCR, biochemical recurrence; 95% CI, 95% confidence interval; y, years.

Abbreviations: HR, hazard ratio; PSA, prostate‐specific antigen.

## 4. Discussion

Lymph node metastasis in patients undergoing RP for clinically localized PCa has long been recognized as an adverse prognostic factor. Accurate identification of nodal involvement may, therefore, have important implications for postoperative risk stratification and treatment selection. Some investigators have advocated extended pelvic lymphadenectomy in place of limited dissection to improve oncological outcomes [1, 2]. However, given the increased complication rates associated with more extensive lymphadenectomy, the potential risks of ePLND may outweigh its benefits, particularly in men with low‐risk disease [7]. Moreover, assessment of the therapeutic value of ePLND is inherently confounded by stage migration and remains difficult to evaluate in the absence of well‐designed prospective trials.

A systematic review and meta‐analysis have demonstrated that BCR after RP is associated with an increased risk of distant metastases, PCa‐specific mortality, and overall mortality [12]. Importantly, these adverse outcomes are largely confined to patients with aggressive disease features, such as a short PSA doubling time and high Gleason score. In the present study, Kaplan–Meier analysis and univariable Cox regression revealed no statistically significant difference in BCR‐free survival between pN0 and pN1 patients, despite a clear numerical trend toward poorer outcomes in the pN1 groups. The absence of statistical significance despite numerically worse BCR outcomes in pN1 patients may reflect the routine use of early adjuvant ADT and salvage radiotherapy in this cohort, which potentially delayed biochemical progression. Therefore, BCR‐free survival in this cohort should be interpreted as being influenced not only by pathological nodal burden but also by early postoperative multimodal treatment interventions. In addition, the relatively small number of pN1 patients may have limited statistical power. Nevertheless, baseline PSA level > 20 ng/mL emerged as the sole independent prognostic factor for BCR‐free survival, underscoring the importance of early disease burden and timely definitive intervention in patients undergoing RP.

In contrast to BCR‐free survival, pathological lymph node positivity was strongly associated with inferior OS. Patients with pN1 disease experienced a nearly threefold increased risk of all‐cause mortality compared with pN0 patients. Despite postoperative multimodal treatment, pathological nodal involvement remained independently associated with inferior long‐term survival, suggesting that pN1 disease reflects not only locoregional tumor spread but also biologically aggressive systemic disease. Notably, despite this adverse prognosis, approximately 74% of the pN1 patients remained alive at 10 years, likely reflecting the beneficial impact of early systemic therapy. Consistent with previous reports demonstrating survival advantages with early hormonal intervention [13], our findings support the routine consideration of early ADT and, when indicated, salvage radiotherapy in patients with nodal metastases. However, cardiovascular events‐related long‐term ADT could not be completely excluded as a contributor to noncancer mortality.

These clinical observations should be interpreted in light of emerging insights into lymph node biology. Mattei et al. elegantly demonstrated that even extended or “super‐extended” lymphadenectomy fails to remove all primary lymphatic drainage sites of the prostate, highlighting the anatomical limitations of surgical clearance alone [6]. More recently, Terasaki and colleagues provided compelling mechanistic evidence that metastatic cancer cells can hijack mitochondria from immune cells within lymph nodes, thereby impairing antigen presentation and cytotoxic immune responses [8]. This immune dysfunction may facilitate systemic disease progression independent of local tumor control. Together, these findings suggest that the prognostic impact of nodal metastasis may reflect not only tumor burden but also disruption of antitumor immunity within the lymphatic microenvironment.

Accordingly, the central challenges in managing node‐positive PCa may not lie solely in the extent of lymph node dissection but rather in developing strategies to identify biologically significant nodal disease and to counteract immune escape mechanisms. Advances in imaging modalities [14], molecular profiling, and immune‐based therapies may enable more precise identification of high‐risk nodal disease and support the development of tailored surgical and adjuvant treatment approaches.

Several limitations of this study warrant consideration. First, the retrospective design and relatively small number of pN1 patients may limit the generalizability. Second, we did not evaluate perioperative morbidity, cost, or operative time associated with PLND. Third, variability in the surgical technique and the individualized application of adjuvant and salvage therapies may have influenced outcomes. Finally, the limited number of PCa‐specific deaths precluded robust analysis of cancer‐specific survival.

## 5. Conclusions

In this long‐term institutional cohort, pathological lymph node positivity was a significant independent predictor of inferior OS in patients undergoing RP for prostate cancer. This finding highlights the clinical importance of pathological nodal involvement and underscores the need for tailored postoperative management strategies, including early systemic and salvage therapies, in patients with lymph node metastases. Future efforts should focus on integrated clinical, radiological, and biological approaches to identify high‐risk nodal disease and to develop immune‐informed treatment strategies that may improve long‐term outcomes.

**Nomenclature**
ADT: Androgen deprivation therapy
BCR: Biochemical recurrence
OS: Overall survival
PCa: Prostate cancer
PLND: Pelvic lymph node dissection
PSA: Prostate‐specific antigen
RP: Radical prostatectomy

## Funding

This clinical research received no specific grant from any founding agency in public, commercial, or nonprofit sectors.

## Conflicts of Interest

The authors declare no conflicts of interest.

## Data Availability

The data that support the findings of this study are not openly available due to reasons of sensitivity and are available from the corresponding author upon reasonable request.
